# Breast cancer-related lymphedema of the upper limb: integrating early surveillance and functional surgery into a synergistic management paradigm

**DOI:** 10.3389/fsurg.2026.1810682

**Published:** 2026-04-20

**Authors:** Tao Wu, Baixin Li, Bingyang Ma, Fuxing Zhao, Yan Li, Zhen Liu

**Affiliations:** 1 Breast Disease Treatment Center, Affiliated Hospital of Qinghai University, Xining, Qinghai, China; 2 Department of Urology, Affiliated Hospital of Qinghai University, Xining, Qinghai, China; 3 Department of Gastrointestinal Surgery, Affiliated Hospital of Qinghai University, Xining, China; 4 Department of Breast Surgery, Peking Union Medical College Hospital, Chinese Academy of Medical Sciences & Peking Union Medical College, Beijing, China

**Keywords:** breast cancer, lymphatic surgery, lymphatic system, lymphedema, quality of life

## Abstract

Breast cancer-related lymphedema (BCRL) of the upper limb is a chronic complication of breast cancer treatment that significantly impairs patient quality of life. Conventional management typically begins only after overt swelling occurs. This reactive approach delays diagnosis, relies on subjective assessment, and prioritizes volume reduction over functional recovery. Consequently, it often misses the optimal window for intervention. Recent advances in objective early-monitoring technologies, such as hyperspectral imaging and bioelectrical impedance analysis, alongside the development of precision functional lymphatic surgery, are now transforming BCRL care. This article details a novel “Precision Collaborative Management” paradigm, which integrates these diagnostic and surgical advances into a synergistic cycle where monitoring informs clinical decisions and surgical outcomes refine subsequent strategies. This paradigm establishes a continuous management pathway encompassing risk screening, early warning, precise intervention, and long-term follow-up across the entire disease continuum. Through sustained exploration in the aforementioned directions, we aim to transform BCRL management from a reactive symptom treatment to a proactive health safeguard, ultimately empowering breast cancer survivors to achieve a comprehensive and high-quality life. The paradigm facilitates a fundamental shift in BCRL clinical practice from delayed symptomatic treatment to early, function-preserving care.

## 1 Introduction

Advances in breast cancer treatment have significantly improved long-term survival. However, a common complication—breast cancer-related lymphedema (BCRL) of the upper limb—substantially impairs survivors' quality of life (1). This chronic, progressive condition results from impaired lymphatic drainage, causing limb swelling, heaviness, fibrosis, compromised shoulder function, increased infection risk, and psychological distress (2). Its incidence varies with treatment extent, ranging from approximately 5% after sentinel lymph node biopsy to 20%–50% following axillary lymph node dissection combined with radiotherapy (3, 4). Onset can occur days to over a decade post-treatment, representing a persistent lifelong burden (5).

For decades, BCRL management has remained predominantly reactive: patients receive diagnosis and begin conservative treatment only after visible swelling appears, with the primary goal of volume reduction. This traditional paradigm suffers from three inherent deficiencies: diagnostic delay [structural damage precedes visible changes (6)], subjective assessment (reliance on arm circumference and patient reports), and limited therapeutic goals (focus on swelling rather than function). Consequently, many patients miss the optimal subclinical window for intervention, progressing to chronic, difficult-to-manage conditions (7). A 2024 systematic review underscored the importance of early screening and assessed standalone technologies such as bioelectrical impedance analysis (BIA) and immediate lymphatic reconstruction (ILR) (8). However, it presented these technologies largely in parallel, without exploring their integration into a dynamic, synergistic clinical system.

To address this gap, we propose a “Precision Collaborative Management” paradigm. This approach integrates early objective monitoring and function-preserving surgery into a feedback-driven closed-loop system: monitoring data guide surgical timing and strategy, while surgical outcomes refine monitoring protocols and risk assessment. Drawing on recent evidence (2024–2026), we elaborate on the two pillars of this paradigm and construct a clinical pathway encompassing screening, risk stratification, precision intervention, and long-term follow-up. Table 1 compares the evolutionary logic and core distinctions between traditional and proposed paradigms. The specific connotations and latest evidence for these two pillars are detailed in the following sections.

**Table 1:** Comparison of traditional reactive paradigm and precision collaborative management paradigm for BCRL of the upper limb.

Dimension	Traditional reactive paradigm	Precision collaborative management paradigm
Timing of intervention	After visible swelling appears (symptom-driven)	Subclinical stage, triggered by objective monitoring data (BIA >10% decline from baseline)
Primary objective	Volume reduction	Function preservation and quality of life
Assessment methods	Arm circumference measurements; subjective symptom reports	BIA for routine screening; HSI/ICG for confirmatory assessment; patient-reported outcome measures
Surgical approach	Delayed therapeutic surgery for advanced stages (LVA/VLNT after conservative failure)	Preventive ILR during ALND; early LVA/VLNT for established BCRL before irreversible fibrosis
Role of allied health	Reactive rehabilitation after swelling occurs	Integrated rehabilitation: preoperative education, postoperative therapy, long-term surveillance and support
Decision-making basis	Empirical judgment based on clinical stage and visible symptoms	Integrated rehabilitation: preoperative education, postoperative therapy, long-term surveillance and support
Care Coordination	Fragmented: surgery, monitoring, and rehabilitation delivered in silos	Integrated: closed-loop monitoring-surgery-feedback with multidisciplinary team coordination
Outcome measures	Limb volume reduction	Functional status (shoulder range of motion), patient-reported quality of life (LYMQOL), compression garment dependency, infection rates, return to daily activities
Long-term follow-up	Episodic, symptom-triggered visits	Continuous, risk-adapted surveillance with individualized intervals (every 3–6 months based on risk profile)

BCRL, breast cancer-related lymphedema; BIA, bioelectrical impedance analysis; HSI, hyperspectral imaging; ICG, indocyanine green lymphography; LVA, lymphovenous anastomosis; VLNT, vascularized lymph node transfer; ILR, immediate lymphatic reconstruction; ALND, axillary lymph node dissection; LYMQOL, Lymphedema Quality of Life Questionnaire.

## 2 Evolution and integration of early objective monitoring technologies

### 2.1 Limitations of traditional diagnostic methods and the concept of the subclinical stage

The diagnosis and monitoring of breast cancer-related lymphedema (BCRL) have traditionally relied on clinical physical examination, such as arm circumference measurement, and patients' subjective symptom reports (6). Although these methods provide important clinical reference points, they have inherent limitations. They lack sensitivity and are subjective, often detecting changes only after the condition has advanced (8). Damage and dysfunction within the lymphatic system occur long before visible or measurable limb swelling appears. This phase, characterized by injury without symptoms or volumetric change, is termed “subclinical lymphedema” (9). Recent studies confirm that this stage represents a critical window during which intervention may reverse or halt disease progression (9). For example, a prospective study found that using the more sensitive bioelectrical impedance analysis (BIA), up to 40% of patients exhibited definitive lymphatic transport abnormalities before meeting traditional arm circumference-based diagnostic criteria (10). This quantitative evidence challenges the diagnostic logic based solely on volumetric changes (8) and underscores the urgent need for more sensitive, objective monitoring tools.

### 2.2 Principles and clinical basis of bioelectrical impedance analysis

Bioelectrical impedance analysis (BIA) quantifies tissue electrical impedance by applying a weak, fixed-frequency alternating current (11). Lymph fluid accumulates mainly in the conductive extracellular space. When fluid increases abnormally, the measured impedance decreases (12). Thus, BIA provides a quantitative measure of subtle changes in limb fluid composition (13). Multiple high-quality studies support its clinical utility. For instance, a multicenter implementation study found that incorporating a portable BIA device into postoperative follow-up advanced the clinical detection of symptomatic lymphedema by over four months on average (14). This approach offers a practical means for early detection and intervention (15). Current clinical application centers on establishing an individualized fluid baseline and tracking its relative changes during follow-up (16). A segmental impedance reduction exceeding 10% from this baseline, measured via single-frequency BIA, is widely regarded as a clinically significant threshold (17). Broader adoption, however, must still address factors such as device cost, operational standardization, and data comparability across different manufacturers (10).

### 2.3 Hyperspectral imaging and ICG lymphography: emerging technologies for tissue characterization

Hyperspectral imaging (HSI) represents an advanced monitoring paradigm that captures reflected light across hundreds of narrow spectral bands. This generates a unique spectral fingerprint for each tissue pixel, allowing non-invasive quantification of biochemical composition, including water, fat, and blood oxygenation (18, 19). A pioneering demonstrated that HSI can produce high-resolution tissue hydration and oxygenation maps in BCRL patients, precisely localizing edematous regions and identifying concomitant microenvironmental alterations such as ischemia (20). This capability offers a novel window into the pathological progression from edema to fibrosis, potentially enabling non-invasive functional staging (18, 20).

Despite its promise, HSI faces significant obstacles to routine clinical adoption. Equipment remains expensive and cumbersome, with current systems costing $50,000–100,000 and requiring dedicated space (21). Data acquisition and analysis algorithms lack standardization, and the correlation between specific imaging parameters and clinical functional outcomes requires validation in large-scale studies (21, 22). Consequently, HSI is currently better suited as a confirmatory tool for high-risk or complex cases identified by BIA, rather than as a first-line screening modality (22).

Indocyanine green (ICG) lymphography offers complementary capabilities through a different mechanism. The technique involves subcutaneous injection of ICG dye, which binds to lymphatic proteins and is transported through lymphatic vessels. Near-infrared fluorescence imaging tracks this movement in real time, providing dynamic visualization of lymphatic vessel contraction and function (23). This allows clinicians to map functional lymphatic vessels, identify sites of obstruction or dermal backflow, and assess the severity of lymphatic dysfunction. ICG lymphography has become invaluable for surgical planning, particularly for procedures such as lymphovenous anastomosis (LVA), where it helps identify optimal vessels for anastomosis and confirm intraoperative patency. Compared to HSI, ICG lymphography offers superior temporal resolution and functional information but is more invasive and requires specialized training.

### 2.4 Integrating monitoring technologies: A Two-tiered risk stratification approach

BIA and HSI serve complementary roles in BCRL risk stratification. BIA functions as a sensitive, low-cost screening tool suitable for routine surveillance of all postoperative patients. It enables early detection of subclinical fluid accumulation through serial impedance measurements. When BIA identifies a progressive decline (e.g., >10% from baseline), HSI can be deployed as a secondary assessment tool. HSI confirms the presence of edema, localizes affected areas, and evaluates tissue characteristics such as oxygenation and fibrosis. This two-tiered approach optimizes resource allocation: BIA provides broad surveillance, while HSI offers detailed characterization for patients at heightened risk.

The complementary strengths of these technologies are summarized in Table 2, which compares their principles, advantages, limitations, sensitivity, cost, and suggested clinical applications.

**Table 2:** Comparison of bioelectrical impedance analysis (BIA) and hyperspectral imaging (HSI) for BCRL surveillance.

Feature	BIA	HSI
Principle	Measures tissue impedance to detect extracellular fluid changes	Captures spectral reflectance to quantify tissue biochemical composition
Advantages	Low cost, portable, easy to use, quantitative, sensitive to subclinical fluid shifts	Non-invasive, provides high-resolution spatial maps, detects tissue ischemia and fibrosis, enables pathological insight
Limitations	Lacks anatomical detail, affected by electrode placement and hydration status, cannot differentiate edema types	Expensive, bulky equipment, complex data analysis, lacks standardized protocols and reference values
Sensitivity/specificity	High sensitivity (∼80–90%) for detecting subclinical lymphedema; moderate specificity	Emerging data; potential for high sensitivity and specificity but requires validation
Cost	Low (device cost ∼$2,000–5,000)	High (device cost ∼$50,000–100,000)
Portability	Portable, suitable for routine clinic use	Currently limited to specialized centers; portable versions emerging
Training requirements	Minimal; brief training for staff	Extensive; requires expertise in image acquisition and interpretation
Suggested clinical use	First-line screening tool for all patients; serial monitoring to detect early fluid changes	Second-line confirmatory tool for high-risk or symptomatic patients; provides anatomical and tissue composition information for surgical planning

BIA, bioelectrical impedance analysis; HSI, hyperspectral imaging.

### 2.5 Construction of the integrated monitoring path

**Figure 1:**
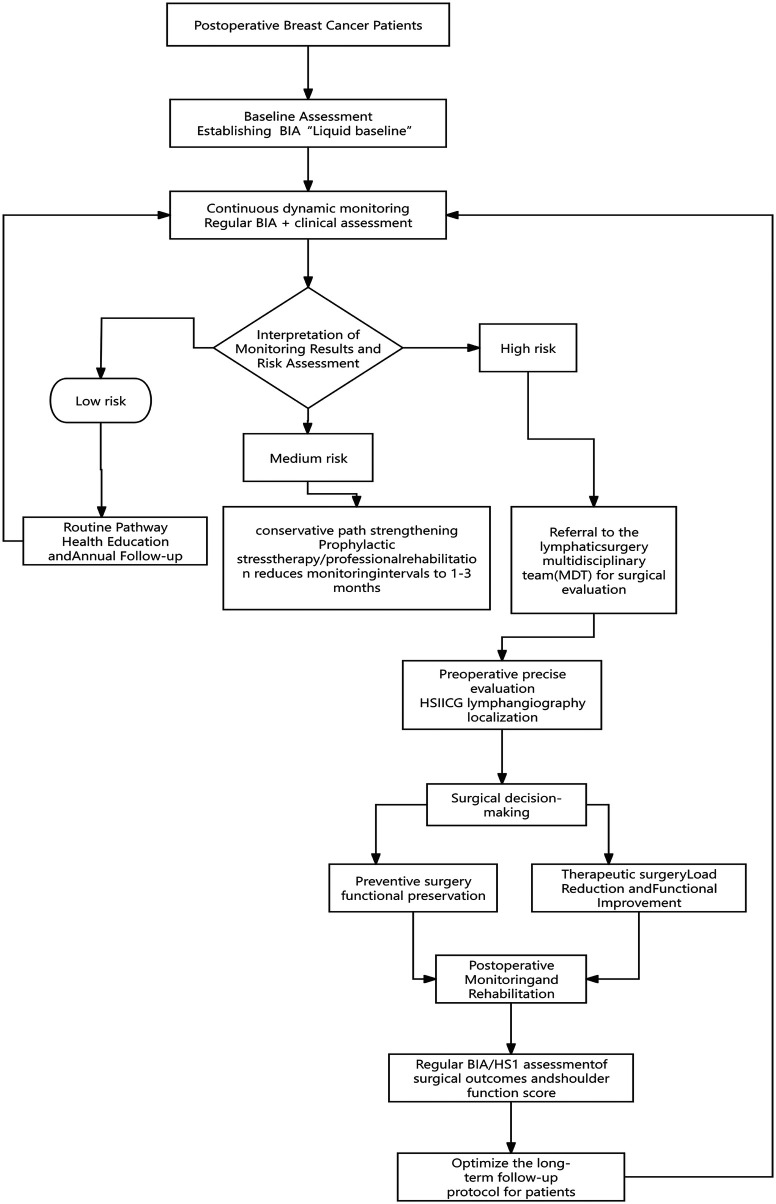
Precision collaborative management pathway for BCRL of the upper limb. Pathway Description: This closed-loop pathway integrates monitoring, risk stratification, surgical intervention, and feedback. The process begins with universal BIA surveillance for all postoperative patients (blue). When BIA shows a sustained decline >10% from baseline, patients enter risk stratification (purple). Low-risk patients continue routine monitoring. Intermediate-risk patients receive intensive conservative therapy led by rehabilitation specialists. High-risk patients are referred to the multidisciplinary team (MDT) for surgical evaluation (green). Preoperative HSI or ICG imaging guides procedure selection—preventive ILR during ALND or therapeutic LVA/VLNT for established BCRL. Postoperative monitoring data feed back into the system (orange), enabling evaluation of surgical efficacy and individualized adjustment of follow-up intervals (every 3–6 months based on risk profile). Rehabilitation teams (PT/OT) provide long-term support including exercise prescription, weight management, skin care, and psychological support. BCRL, breast cancer-related lymphedema; BIA, bioelectrical impedance analysis; HSI, hyperspectral imaging; ICG, indocyanine green lymphography; MDT, multidisciplinary team; ILR, immediate lymphatic reconstruction; ALND, axillary lymph node dissection; LVA, lymphovenous anastomosis; VLNT, vascularized lymph node transfer; PT, physiotherapist; OT, occupational therapist.

Based on the complementarity of these techniques, we propose a three-tiered integrated active surveillance pathway (Figure 1). This pathway progresses from universal screening to precision screening and finally to risk stratification. The goal is to achieve efficient and precise risk management (24). Stage 1: all patients were established postoperatively with BIA baseline and included in routine oncology follow-up; Low-cost, high-efficiency longitudinal fluid component monitoring (16). Stage 2: Initiation of a secondary assessment in patients who show a progressive decline in BIA (a sustained relative impedance reduction of >10% from their postoperative baseline), which is widely regarded as a clinically significant threshold indicative of extracellular fluid accumulation and subclinical lymphedema, as supported by multiple studies, or who present with new-onset symptoms. At the core of this stage is the introduction of imaging tests such as HSI to confirm abnormalities, anatomic localization, and evaluation of tissue texture, achieving the transformation from “A bnormal signals” to “A bnormal images” (25). Stage 3: combining objective surveillance data with static clinical high-risk factors (surgical extent, radiotherapy history, high BMI, etc.), patients were dynamically classified as low, medium, and high risk (26). Intensive education was initiated in low-risk individuals. For intermediate-risk individuals, an intensive non-surgical intervention led by specialized lymphedema therapists or physiotherapists was initiated, and surveillance intervals were shortened. This intervention comprises a structured program including manual lymphatic drainage (MLD), multi-layer compression bandaging, and tailored exercise prescription (progressive resistance exercises and range-of-motion activities), typically initiated with weekly sessions for 4–6 weeks, followed by reassessment to evaluate response and adjust the plan. High-risk individuals were immediately referred to a lymphosurgical multidisciplinary team for surgical evaluation (27). This pathway ensures a precise match between medical resources and patient risk (28).

## 3 Precision development of function-oriented surgery

Surgical interventions for BCRL can be broadly categorized into preventive and therapeutic strategies. Preventive surgery, primarily immediate lymphatic reconstruction (ILR), is performed concurrently with axillary lymph node dissection to reduce the risk of developing lymphedema. Therapeutic surgery, including lymphovenous anastomosis (LVA) and vascularized lymph node transfer (VLNT), aims to restore lymphatic drainage in patients with established BCRL. The following sections detail the evidence, patient selection criteria, and outcomes for each approach.

### 3.1 Maturity of preventive surgery

Immediate lymphatic reconstruction (ILR) is a microsurgical technique performed during axillary lymph node dissection for breast cancer. It involves anastomosing transected lymphatic vessels to nearby veins to re-establish lymphatic drainage and prevent lymphedema (29). ILR has evolved from an exploratory procedure into a preventive strategy supported by relatively high-level evidence. Multiple systematic reviews and meta-analyses have demonstrated that ILR reduces the absolute risk of symptomatic lymphedema by 15–20 percentage points in high-risk patients undergoing ALND, corresponding to an approximate two-thirds reduction in relative risk (30, 31). These pooled analyses primarily comprise single-center cohort studies, which, while consistent in their findings, warrant confirmation through multicenter randomized trials.

Patient selection is critical for optimizing ILR benefit. Current evidence supports offering ILR to patients undergoing ALND, particularly those with additional risk factors: planned regional radiotherapy, preoperative BMI >30, tumor located in the lateral quadrant, or prior ipsilateral axillary surgery (31, 32). The presence of multiple risk factors strengthens the indication for ILR. Contraindications may include advanced age, significant comorbidities precluding prolonged surgery, or patient preference against additional microsurgical procedures.

### 3.2 Preliminary exploration of combined flap transplantation and functional reconstruction

The surgical paradigm has evolved. The goal is no longer simply to prevent lymphedema. The higher-order objective is now to proactively preserve postoperative shoulder function (32). This philosophy is embodied in a combined surgical procedure. It integrates immediate lymphatic reconstruction (ILR) with a prophylactic vascularized pedicled flap transfer, such as the lateral thoracic artery perforator (LTAP) flap (33). The design rationale for this procedure extends beyond physiological reconstruction. By providing soft tissue padding in the axilla, the transferred flap can reduce postoperative dead space and mitigate scar contracture, thereby directly improving shoulder range of motion (34). Preliminary clinical reports indicate that patients receiving the combined procedure show significantly greater improvement in active shoulder abduction angle at six-month follow-up than those undergoing traditional surgery (34). These findings suggest a deepening integration of lymphatic surgery with functional reconstructive surgery (31). While its long-term efficacy and optimal indications require further validation, this approach clearly points the way forward: a successful prophylactic procedure should aim not merely to reduce the incidence of one complication but to help shape a more functional upper limb for the patient (31).

The combined ILR and flap transfer procedure represents an innovative concept but currently rests on limited evidence. Preliminary reports are single-center, small-sample studies without control groups (34). While early functional outcomes are encouraging, these findings must be interpreted cautiously. Long-term benefits, optimal patient selection, and comparative effectiveness against ILR alone require rigorous evaluation through multicenter prospective studies. This approach should currently be considered experimental and offered only within research settings or by highly experienced teams with appropriate patient consent.

### 3.3 Lymphatic venous anastomosis and vascularized lymph node transplantation

For patients with established BCRL, physiologic surgical procedures such as lymphovenous anastomosis (LVA) and vascularized lymph node transfer (VLNT) aim to restore lymphatic drainage and reduce limb volume. A two-year follow-up from a multicenter randomized controlled trial showed that, compared to complex decongestive therapy alone, patients undergoing LVA achieved significant limb volume reduction and reduced daily compression garment use by an average of 68% (35). This patient-centered functional outcome underscores the value of early surgical intervention guided by objective assessment. VLNT, which transfers healthy lymph nodes to the affected limb, has also shown efficacy in multiple cohort studies, though randomized evidence remains limited (36).

The evidence base for LVA is strengthened by the aforementioned RCT, but most VLNT studies are single-center case series with small sample sizes, limiting generalizability. Both procedures demonstrate better outcomes when performed in early-stage (International Society of Lymphology stage I–II) lymphedema, highlighting the importance of early detection (37).

Patient selection for therapeutic surgery depends on several factors. Ideal candidates for LVA have identifiable functional lymphatic vessels on preoperative ICG lymphography, typically in early-stage disease without extensive dermal backflow (38). VLNT may be preferred in patients with advanced fibrosis or those lacking suitable recipient vessels for LVA. Additional considerations include patient goals (volume reduction vs. functional improvement), willingness to adhere to postoperative compression, and access to specialized surgical teams.

Long-term outcomes beyond two to three years are still emerging. Available data suggest sustained volume reduction and improved quality of life, but graft function durability and potential donor-site morbidity require longer follow-up. Multicenter prospective registries and randomized trials comparing LVA, VLNT, and conservative management are urgently needed to establish comparative effectiveness and inform treatment algorithms.

### 3.4 Importance of patient-centered functional outcomes

As surgical objectives evolve toward functional preservation, patient-reported outcome measures (PROMs) must become primary endpoints rather than secondary considerations. Accordingly, the system for evaluating efficacy must also advance (37). Although the traditional core metric of percentage reduction in limb volume remains relevant, it is now insufficient for fully capturing an intervention's impact on patient life (38). Patient-centered functional and quality-of-life indicators—such as Lymphedema Quality of Life Questionnaire (LYMQOL) scores, shoulder range of motion, pain scores, and dependence on assistive devices like compression garments—have gained critical importance (39). These measures correlate directly with patients' daily activity capacity and psychosocial status (37). Reducing reliance on compression garments improves comfort, body image, and social confidence (36).Future clinical trials and practice will therefore inevitably integrate volumetric metrics with functional and quality-of-life indicators for a comprehensive assessment (37, 40). This trend requires clinical researchers to develop and employ more standardized, sensitive patient-reported outcome measures (41).

## 4 Establishing a collaborative management system for monitoring and surgery

Early objective monitoring and function-oriented surgery are not two separate steps in the management process. As this chapter will show, the two can only be closely integrated and mutually validated. Only by using the monitoring results to guide surgical decision-making in real time, and then using the surgical results to verify and optimize the monitoring scheme, can a continuous improvement cycle be formed. This is the key to achieving “Precision collaborative management” (31).

### 4.1 Dynamic feedback between monitoring and surgery

The traditional linear management paradigm is ill-suited to BCRL, which is a chronic, dynamic disease (42). The collaborative logic of surveillance and surgery is rooted in three imperatives. First, decision-making must be precise. The success of function-preserving surgery depends heavily on correct timing and indication. Clinical experience or rough staging alone cannot meet this requirement. Instead, continuous, objective monitoring data must provide decision triggers and justify the chosen protocol (43). Second, there is a need for objective evaluation of efficacy. The success of surgical intervention can not be judged only by subjective feelings. Regular postoperative BIA and HSI assessments provide quantifiable, visual evidence of surgical outcome, which is the basis for evaluating long-term surgical efficacy and technical improvement (36). Finally, the need for individualized management. Each patient responds differently to treatment. The core of the system lies in the real-time feedback of postoperative monitoring data, which is used to dynamically adjust the follow-up time and rehabilitation protocol of this patient, realize personalized management, and improve the quality of life, thus, the chronic disease management paradigm is upgraded from a static scheme to a dynamic adaptation process (44).

### 4.2 Path diagram and step-by-step explanation

Based on the above logic, we constructed the following collaborative management clinical pathway (Figure 1) to provide a panoramic view of the practice flow of this paradigm (27). Continuous monitoring with BIA-based tools serves as the engine of this closed-loop system, continuously generating data that dynamically categorizes all patients and ensures those at different risk levels follow appropriate pathways. Identification of high-risk patients is no longer based on late-stage symptoms, but on objective data. The key referral trigger is a sustained BIA reduction of >10% from postoperative baseline, which automatically generates an alert in the electronic health record and prompts referral to the lymphosurgical multidisciplinary team (16). Additional triggers include new-onset symptoms reported by patients or clinical findings during routine follow-up.

At multidisciplinary team meetings, data from BIA, HSI, and ICG lymphography are reviewed collectively. Surgeons, radiologists, and rehabilitation specialists discuss each case to reach consensus on the appropriate intervention. Decision points include: (1) whether to proceed with surgical intervention, (2) which procedure is most suitable based on anatomical and functional assessment, and (3) timing of surgery relative to ongoing adjuvant therapies.

For patients proceeding to surgery, preoperative HSI or ICG images serve as “surgical maps,” enabling evidence-based planning for preventive or therapeutic procedures. Postoperative monitoring data are fed back into the system, allowing the team to evaluate surgical outcomes and adjust follow-up protocols accordingly.

Postoperative quantitative results serve two purposes: they evaluate surgical efficacy and inform individualized follow-up. This individualization is a collaborative process between surgeons and rehabilitation teams. Based on BIA and functional assessment data, physiotherapists and occupational therapists design a long-term plan that may include exercise programs, weight management, skin care, and psychological support. Follow-up frequency (every 3–6 months) is tailored to the patient's risk profile and recovery trajectory (26).

### 4.3 Barriers in technology, process, and multidisciplinary collaboration

Although the idea of this management system is very clear, there are still some practical difficulties to be applied to clinical practice. First, existing technical tools require further refinement. BIA devices from different manufacturers lack standardized measurement protocols and reference values, complicating data comparison across centers and longitudinal follow-up (13). HSI technology remains expensive and operationally complex; its high cost (typically $50,000–100,000) and need for specialized training limit its use to academic centers (21). Moreover, seamless data integration between BIA, HSI, and electronic health records is currently lacking, hindering real-time clinical decision-making and the development of large-scale databases for algorithm training. These technical barriers must be addressed through industry collaboration and professional society guidelines to enable widespread adoption. Second, there are challenges in process re-engineering. Current hospital workflows are mostly linear, with distinct phases for diagnosis, treatment, and follow-up. The proposed system requires establishing regular monitoring schedules, defining clear data-driven early warning and referral criteria, and creating unified postoperative follow-up protocols. This represents a fundamental transformation of internal hospital workflows (28).Third, barriers to multidisciplinary collaboration persist. The effectiveness of this system depends heavily on close coordination among oncology surgeons, plastic and lymphatic surgeons, radiologists, rehabilitation therapists, and nursing teams. Breaking down departmental silos and establishing efficient communication and shared decision-making processes remain major challenges. Without structured collaboration, the closed-loop monitoring-surgery-feedback cycle cannot function effectively.

Finally, patient compliance forms the foundation of this paradigm. The success of long-term monitoring depends on patients understanding and adhering to regular follow-up schedules. This requires continuous, effective patient education and support systems to improve treatment adherence (42). Strategies such as mobile health reminders, peer support programs, and simplified follow-up procedures may help address this challenge. Several strategies can improve patient adherence. First, structured education at the time of surgery should explain surveillance rationale, BIA measurements, and benefits of early intervention, reinforced by visual aids and take-home materials. Second, digital health tools—including mobile apps with automated appointment reminders, secure messaging, and graphical BIA trend displays—can empower patients to actively participate in their care. Third, peer support programs connecting new patients with long-term BCRL survivors provide emotional support and practical guidance. Fourth, simplifying follow-up by coordinating visits with oncology appointments, offering telehealth options, and minimizing wait times reduces practical barriers. Finally, addressing financial concerns through insurance counseling and assistance programs alleviates economic barriers to regular follow-up.

### 4.4 Data platform, consensus and payment paradigm

In view of the above practical difficulties, we need to plan and design in advance from the perspective of system construction. The first task is to build a unified management platform that can integrate all information such as BIA, HSI, clinical records and patient feedback. This is the cornerstone of using data to accurately guide clinical decision-making (10).

There is an urgent need for clear standards of practice, led by authoritative professional societies, to be established—for example, how to define the warning value of monitoring data, how to choose the best time for surgery, and how to structure multidisciplinary team collaboration. With clear guidelines, clinical practice can be informed (45).

The economic feasibility of this collaborative paradigm depends on sustainable payment models. The current fee-for-service approach, which reimburses individual procedures and visits, fails to capture the value of preventive monitoring, multidisciplinary coordination, and long-term functional outcomes. This misalignment creates financial disincentives for implementing the comprehensive pathway we propose (46).

Several alternative payment models warrant exploration. Bundled payments, which provide a single reimbursement covering all care related to BCRL prevention and management over a defined episode or time period, could align incentives across providers and encourage coordination. For example, a bundled payment for the first two years following axillary dissection could cover BIA surveillance, MDT consultations, and ILR if indicated.

Value-based payment models, which tie reimbursement to functional outcomes and patient-reported quality of life, represent another promising direction. Such models would reward providers for achieving meaningful results—such as maintaining shoulder range of motion or avoiding compression garment dependency—rather than simply delivering services.

From a health system perspective, the cost-effectiveness of this paradigm requires formal evaluation. While upfront costs for monitoring devices and surgical procedures are higher than traditional care, potential long-term savings from reduced chronic lymphedema treatment, fewer infections, and improved productivity may offset these investments. Pilot studies with embedded economic analyses are needed to generate local data for payers and policymakers.

International experiences can inform payment reform. In countries where preventive lymphatic surgery and surveillance programs have been integrated into national health insurance, early evidence suggests favorable cost-effectiveness ratios. Cross-country learning collaborations could accelerate adoption of sustainable payment models globally.

**Figure 2:**
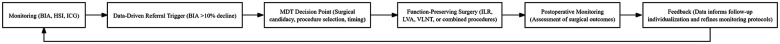
The core feedback loop of precision collaborative management.

This simplified diagram illustrates the essential closed-loop logic of the proposed paradigm. Continuous monitoring (BIA, HSI, ICG) generates objective data. A sustained BIA decline >10% triggers referral to the multidisciplinary team (MDT). The MDT makes key decisions regarding surgical candidacy, procedure selection, and timing. Function-preserving surgery (ILR, LVA, VLNT, or combined procedures) is performed. Postoperative monitoring assesses surgical outcomes. These results feed back into the system, enabling individualized follow-up planning and continuous refinement of monitoring protocols. This cycle ensures that care is dynamically adapted to each patient's response and evolving risk profile (Figure 2).

## 5 Discussion

This chapter aims to examine the core values of the “precision collaborative management paradigm”, analyze its basis, and confront its current limitations, so as to position the contribution of this review in a broader academic and practical context.

### 5.1 Early objective monitoring: progress and unresolved challenges

Objective monitoring technologies—particularly BIA and HSI—have shifted the paradigm toward detecting subclinical BCRL. BIA enables early identification of extracellular fluid accumulation, with evidence suggesting it can advance diagnosis by over four months compared to traditional methods (14). This aligns with the fundamental principle that earlier intervention improves outcomes.

However, critical gaps remain. First, the evidence base for BIA is predominantly derived from single-center studies with heterogeneous protocols, limiting generalizability (13). Second, the widely used 10% threshold for clinical significance, while supported by some studies (17), lacks prospective validation across diverse populations and clinical settings.

HSI represents a technological leap, offering non-invasive insights into tissue composition and microenvironmental changes (20). Yet its clinical utility remains largely investigational. Current evidence consists of small, proof-of-concept studies without standardized acquisition protocols or validated diagnostic criteria (21). The correlation between HSI parameters and functional outcomes—such as shoulder mobility or patient-reported quality of life—has not been established.

Publication bias is a concern, as studies reporting positive findings are more likely to be published than those showing null results. Additionally, most studies originate from high-resource academic centers, raising questions about applicability in community settings.

The path forward requires multicenter validation studies, standardized protocols endorsed by professional societies, and rigorous investigation of the relationship between imaging biomarkers and clinically meaningful outcomes.

### 5.2 Function-Oriented surgery: evidence, limitations, and evidence gaps

The evolution of surgical objectives from volume reduction to functional preservation represents a significant conceptual advance. However, the evidence base supporting different surgical strategies varies considerably, and critical gaps remain.

Table 3 summarizes the current evidence level, key limitations, and evidence gaps for the main surgical approaches discussed in this review. Across all surgical approaches, several common limitations emerge. First, the lack of standardized outcome measures hinders cross-study comparison and meta-analysis. While limb volume reduction remains the most frequently reported endpoint, functional outcomes (shoulder range of motion, upper extremity disability) and patient-reported outcome measures are inconsistently collected and reported (40). Second, follow-up duration is generally insufficient to assess long-term durability of surgical benefits and late complications, such as donor-site morbidity or recurrence of edema. Third, most studies originate from high-volume centers with specialized expertise in microsurgery and lymphedema care, raising questions about generalizability to broader surgical practice and community settings.

**Table 3:** Evidence profile of surgical strategies for BCRL.

Surgical strategy	Evidence level	Key limitations	Evidence gaps
ILR (preventive)	Multiple systematic reviews and meta-analyses of cohort studies	Predominantly single-center; no published randomized controlled trials; variable patient selection criteria; short to medium-term follow-up	Long-term (>5 years) durability; optimal patient selection criteria; comparative effectiveness vs. surveillance alone; impact on patient-reported outcomes
LVA (therapeutic)	One multicenter randomized controlled trial with 2-year follow-up; multiple cohort studies	RCT evidence limited to single trial; lack of blinding; short follow-up; heterogeneous outcome measures across studies	Durability beyond 2–3 years; comparative effectiveness vs. VLNT; predictors of response; optimal timing of intervention
VLNT (therapeutic)	Multiple single-center case series;no randomized trials	No RCTs; small sample sizes (typically <50 patients); heterogeneous techniques (donor sites, recipient sites) and outcome measures; lack of standardized assessment protocols	Comparative effectiveness vs. LVA; optimal donor site (groin vs. omentum vs. supraclavicular); long-term donor-site morbidity; patient selection criteria
Combined ILR + flap	Single-center pilot studies with small samples	No control groups; very small samples (typically <20 patients); short follow-up (<1 year); lack of standardized protocols	Efficacy compared to ILR alone; optimal patient selection; safety profile; cost-effectiveness; long-term functional outcomes

Potential biases warrant explicit acknowledgment. Publication bias likely favors studies reporting positive outcomes, particularly for newer procedures such as combined ILR-flap reconstruction. Selection bias is inherent in non-randomized studies, where healthier patients or those with less severe disease may be preferentially offered surgery. Measurement bias may arise from lack of blinding in outcome assessment, particularly for subjective endpoints such as patient-reported symptoms or quality of life. The absence of a core outcome set for BCRL surgery further complicates evidence synthesis and increases the risk of selective outcome reporting (44).

These limitations underscore several urgent research priorities. Rigorous, multicenter randomized trials with long-term follow-up (minimum five years) are needed to establish comparative effectiveness of surgical strategies against each other and against conservative management. Such trials must incorporate standardized outcome measures, including limb volume, functional status, patient-reported quality of life, and cost-effectiveness. Development and international consensus on a core outcome set for BCRL surgery would facilitate evidence synthesis and reduce reporting bias (44). Until such evidence accumulates, clinical decision-making must balance the promising but limited available data with individual patient preferences and local expertise.

### 5.3 System implementation: From concept to reality

The proposed collaborative paradigm is logically coherent and conceptually appealing, envisioning a future of data-driven, personalized chronic disease management. However, its translation into clinical practice faces substantial barriers that extend beyond individual technologies to fundamental healthcare system challenges. Process-level barriers include the misalignment between current linear care pathways and the iterative monitoring-surgery-feedback loop required by this paradigm. Implementing regular surveillance, data-driven referral triggers, and coordinated MDT decision-making requires fundamental redesign of hospital workflows (27).

Professional barriers include the need for interdisciplinary collaboration, which remains elusive in many institutions. Effective implementation requires shared ownership among surgeons, rehabilitation specialists, radiologists, and nursing teams, a cultural shift from traditional siloed practice.

Patient-level barriers include variable health literacy, competing priorities during cancer survivorship, and financial constraints. Even well-designed surveillance programs fail if patients do not adhere to follow-up schedules (45).System-level barriers include payment models that reward volume over value and the lack of infrastructure for integrating multimodal data across devices and electronic health records. Addressing these barriers requires a coordinated research agenda. Based on the preceding analysis, we propose the following prioritized future research directions: Multicenter randomized trials evaluating ILR vs. surveillance in high-risk populations, and LVA vs. conservative management in early-stage BCRL, with long-term (>5 years) follow-up and standardized outcome measures including function, quality of life, and cost-effectiveness. Development and validation of standardized imaging biomarkers, linking HSI and ICG parameters to clinically meaningful outcomes, enabling non-invasive staging and treatment response monitoring. Artificial intelligence decision support systems integrating multimodal data (BIA, imaging, clinical risk factors) to provide dynamic risk predictions and personalized intervention recommendations. Implementation science research identifying barriers and facilitators across diverse healthcare settings, and developing adaptable implementation strategies tailored to local resources and contexts. Health economics research evaluating cost-effectiveness of the collaborative paradigm and informing sustainable payment models, including bundled payments and value-based reimbursement.

Enhancing translational relevance across diverse healthcare settings requires deliberate adaptation of this paradigm to local resources and contexts. In high-resource academic centers, full implementation with all monitoring technologies (BIA, HSI, ICG) and surgical options (ILR, LVA, VLNT, combined flaps) may be feasible. In community hospitals or low-resource settings, a stepped implementation approach can be considered:

Tier 1 (Essential): Universal BIA surveillance, structured patient education, and clear referral pathways to regional lymphedema specialists or surgical centers.
Tier 2 (Intermediate): Addition of telehealth-based MDT consultations, remote monitoring support via mobile applications, and peer support programs.
Tier 3 (Comprehensive): Full implementation with HSI/ICG imaging, on-site MDT, and all surgical options, supported by bundled payment models and integrated data platforms.

Cross-setting learning collaboratives can facilitate knowledge exchange and adaptation of best practices. Implementation research should identify context-specific barriers and facilitators, develop tailored implementation toolkits, and evaluate outcomes across diverse settings to ensure equitable access to advances in BCRL care.

Through sustained investigation in these priority areas, the vision of transforming BCRL management from reactive symptom treatment to proactive health preservation can be realized.

## 6 Conclusions

This review systematically integrates evidence from 2024 to 2026, demonstrating that breast cancer-related lymphedema management is at a critical juncture. The traditional reactive paradigm, focused on volume reduction after swelling appears, no longer meets patients' needs. We propose a “Precision Collaborative Management Paradigm” that establishes a dynamic “monitoring-surgery-feedback” cycle. This approach enables early risk-driven intervention through continuous objective monitoring and prioritizes functional preservation in surgical decision-making, thereby realizing truly individualized long-term care.

This paradigm is not a mere enumeration of existing technologies, but rather aims to provide a novel systematic thinking paradigm. It emphasizes that future advancements will increasingly stem from deep, organic synergies among monitoring, surgical, rehabilitation, and data systems, rather than isolated breakthroughs in individual technologies.

Based on this analysis, we propose the following specific and pressing directions for future research. Prospective, multicenter studies are needed to rigorously compare long-term functional outcomes, patient quality of life, and health economic costs between the collaborative closed-loop pathway proposed here and conventional management strategies (47). Spectral data from techniques such as HSI require in-depth investigation. Analytical methods like radiomics, the high-throughput extraction of quantitative features from medical images that are not visible to the naked eye, should be employed to identify quantitative imaging biomarkers that correlate strongly with lymphatic function, tissue fibrosis, and surgical outcomes, thereby enabling truly non-invasive and precise staging. Artificial intelligence and machine learning should be leveraged to develop intelligent decision support systems capable of integrating multimodal monitoring data, clinical information, and prior evidence. These systems must provide clinicians with dynamic risk predictions and personalized intervention recommendations (48). Research should investigate the barriers to and facilitators of implementing this paradigm across diverse healthcare settings. Studies should also explore innovative payment paradigms that incentivize multidisciplinary collaboration and preventive care, thereby informing policy development (49).

Prospective, multicenter studies are urgently needed to compare long-term functional outcomes, patient quality of life, and health economic costs between the collaborative closed-loop pathway and conventional management. Specifically, randomized trials should evaluate ILR vs. no ILR in high-risk populations, and LVA vs. conservative therapy in early-stage BCRL. Long-term follow-up beyond five years is essential to assess durability of surgical benefits and late complications.

Through sustained exploration in these directions, we aim to transform BCRL management. The goal is to shift from reactive symptom treatment to proactive health preservation. Ultimately, this will empower breast cancer survivors to achieve a comprehensive and high-quality life.
